# Physical Origin of Early Failure for Contaminated Optics

**DOI:** 10.1038/s41598-018-37337-5

**Published:** 2019-01-24

**Authors:** Andrew Brown, David Bernot, Albert Ogloza, Kyle Olson, Jeff Thomas, Joseph Talghader

**Affiliations:** 1 University of Minnesota Twin Cities, Minneapolis, MN 55455 USA; 2 Penn State Electro-Optic Center, Freeport, PA 16229 USA; 3 Schafer, Arlington, VA 22203 USA

## Abstract

Laser-Induced optical breakdown often occurs unexpectedly at optical intensities far lower than those predicted by ultra-short pulse laser experiments, and is usually attributed to contamination. To determine the physical mechanism, optical coatings were contaminated with carbon and steel microparticles and stressed using a 17 kW continuous-wave laser. Breakdown occurred at intensity levels many orders of magnitude lower than expected in clean, pristine materials. Damage thresholds were found to strongly follow the bandgap energy of the film. A thermal model incorporating the particle absorption, interface heat transfer, and free carrier absorption was developed, and it explains the observed data, indicating that surface contamination heated by the laser thermally generates free carriers in the films. The observed bandgap dependence is in direct contrast to the behavior observed for clean samples under continuous wave and long-pulse illumination, and, unexpectedly, has similarities to ultra-short pulse breakdown for clean samples, albeit with a substantially different physical mechanism.

## Introduction

Five decades of research have created optics and coatings capable of operating at average powers of hundreds to thousands of kWcm^−2^ in controlled environments while maintaining consistent optical properties^1–3^. Unfortunately for optical designers, the world is not a clean room. Contamination, whether from manufacturing, environmental, or human sources is all but inevitable. Contaminants can easily lower damage thresholds by orders of magnitude, meaning that the practical damage threshold is usually limited by cleanliness rather than intrinsic material’s structure or film absorption^1,4–6^. Further complicating the issue is that the majority of laser damage experiments use carefully controlled laboratory conditions with short-pulsed lasers focused to small spots on clean, pristine materials. Outside of the laboratory, optical failure occurs under radically different conditions. Understanding how surface contamination initiates damage and determining what can be done to mitigate its effects are crucial for designing optics that can survive in typical (dirty) conditions.

This work finds that the physical mechanism behind contamination-induced breakdown is thermal free carrier generation and subsequent absorption. This mechanism predicts a strong bandgap dependence for breakdown, which is clearly observed in experimental testing. Interestingly, one gains little or no improvement in breakdown characteristics when using protective layers of high bandgap material with typical optical coating thicknesses, a result that can be mathematically predicted by calculating the volume of the optical substrate that is heated by contaminant evaporation. The experimental findings of the bandgap dependence demonstrates that large bandgap materials are more reliable when used in high power applications.

## Materials and Methods

The optics selected for this study were 1″ fused silica superpolished optical flats with.

Low-absorption metal oxide coatings deposited using ion beam sputtering (IBS) [ATFilms]. Both half-wave (λ/2) and high reflectivity (HR) distributed Bragg reflectors (DBRs) were fabricated. Each λ/2 sample was one of five types, consisting of a single half-wave layer of titania, tantala, hafnia, alumina, or silica. Each HR sample was composed of 20–44 λ/4 pairs of titania-silica, niobia-silica, tantala-silica, or hafnia-silica. Both the λ/2 and HR samples were designed for a center wavelength of 1064–1070 nm, corresponding to the emission wavelengths of Nd^3+^:YAG solid-state and ytterbium-doped fiber lasers. Prior to contamination, optical absorption values of these samples were measured to be between 0.7 ppm and 35 ppm using photothermal common-path interferometry (PCI)^7–10^.

**Figure 1:**
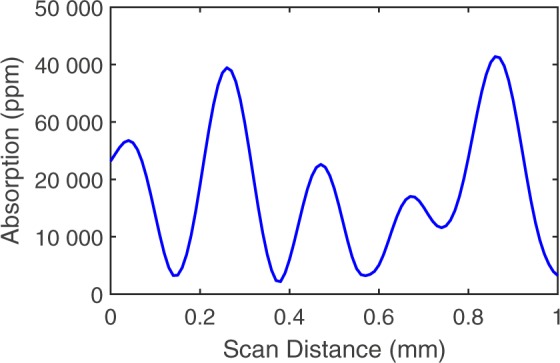
A typical PCI scan across the surface of a carbon contaminated sample.

Large absorbing particles are known to dramatically reduce damage thresholds^6,11^. Carbon-based contamination, as is often encountered in routine handling or environmental exposure, is particularly problematic^1^. For this reason, carbon microparticles were selected as the primary test contaminant. These particles had an average size of 7 µm and had very high optical opacity. The optics were contaminated using a suspension of the carbon microparticles in isopropyl alcohol with a density of 0.07 g/mL. A drop 0.15 mL of the suspension was placed onto the optics, briefly allowed to settle, and then dried with a compressed nitrogen flow. The absorption of the surface after contamination was measured using PCI and averaged from thousands to tens of thousands of ppm, see Fig. 1. The much larger spot of the laser used in the damage testing as compared to the PCI spot size (56 µm), effectively averages out the small local absorption variations. Stainless steel microparticles of diameter 35–40 µm were also used as a contaminant in some tests since metallic particles are a frequently encountered contaminant created during the assembly of optical systems^4,11^.

Laser damage testing was conducted at Penn State’s Electro-Optics Center using a 17 kW CW Ytterbium Doped IPG photonics (IPG YLS-17000) fiber laser at 1070 nm to illuminate samples. The high power of the laser permitted testing of several MWcm^−2^ irradiances with spot sizes on the order of a millimeter. Weaker lasers must sacrifice spot size for irradiance, and can often only illuminate diameters of tens of µm. The larger spot sizes used in our testing are advantageous as they represent a better average of typical regions of an optic, with many defects or contaminants being illuminated. Before testing the power of the laser, the beam size and shape were measured using a Primes PM100 power meter and a Primes FM120 focus monitor. The output of the laser was approximately Gaussian with an M^2^ less than 6, and was focused to produce a spot sizes varying from 1 mm to 400 µm depending on the required irradiance. The vast majority of testing occurred at 1 mm as this provided a large illuminated spot yet still allowed irradiances up to 3 MWcm^−2^ to be tested, which was sufficient to damage all but the large bandgap half-wave coatings. This was reduced to 400 µm as required to test at the highest irradiances. The Primes power meter was used as a water cooled beam dump to catch all the reflected or transmitted light off the samples. For safety the laser was fired remotely from the adjacent room, protected by OD absorbing filters over the windows. A large vacuum system was placed nearby the sample to filter out any airborne contaminants.

**Figure 2:**
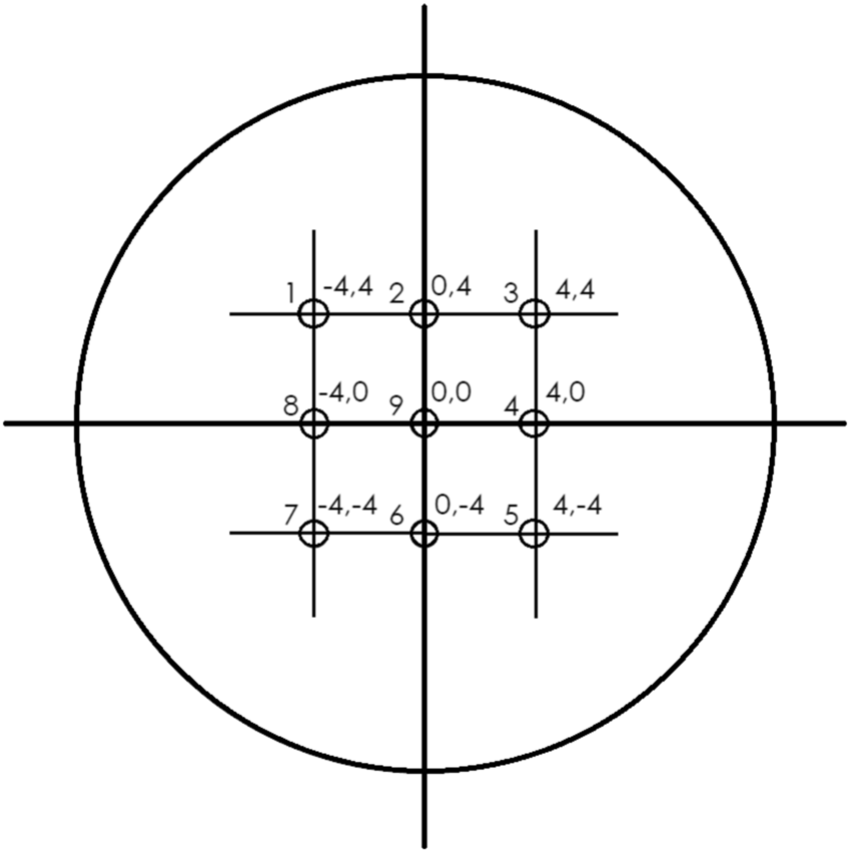
Test grid used during laser damage exposure. Each location is spaced 4 mm apart to maximize the use of each sample but also to prevent any influence from previous exposures.

Testing of samples was performed using a 3 × 3 grid with each location spaced 4 mm apart to prevent any previous tests from affecting adjacent test locations, see Fig. 2. Each location was exposed once, stepping up the laser power at each new location until failure occurred. A thermal camera monitoring the sample during testing provided information about the approximate surface temperature of the optic and could qualitatively tell how close a sample was to failure. Once a failure had occurred, irradiances above and below it were tested with the goal of finding an irradiance that caused damage for 50% of exposures. Variations in the contamination density cause differences in the damage threshold from location to location. For this reason we report the minimum damaging irradiance and the maximum survived irradiance as this better captures the true spread of the data.

Samples with silica protective capping layers were also included in the contaminated testing. These experiments were designed to determine if early failure could be mitigated by the application of a thin protective cap composed of a material that was extremely robust in resisting contamination-induced breakdown. These silica cap layers were of thickness equal to 3 to 15 half-waves (1.1–5.5 µm) and were deposited over tantala-silica DBRs. They were contaminated with carbon and tested in the same fashion as the normal DBRs and λ/2 coatings. To test very thick protective coatings, carbon was applied to the back surface of the silica substrate and the sample was illuminated from the backside, effectively using the substrate’s thickness as a capping layer.

An additional test was performed to determine if short-wavelength photons thermally emitted from the superheated particles would contribute to free-carrier induced laser damage. In addition to testing contaminated coatings, pristine coatings were tested for their 1.07 µm laser damage thresholds while being simultaneously irradiated with a 40 mW 224 nm deep-UV laser. The UV laser beam was focused as tight as possible and was measured at less than 5 µm wide using the knife edge technique. This experiment was done to test if UV photons above the bandgap might help initiate that laser damage process^12,13^.

## Results

**Figure 3:**
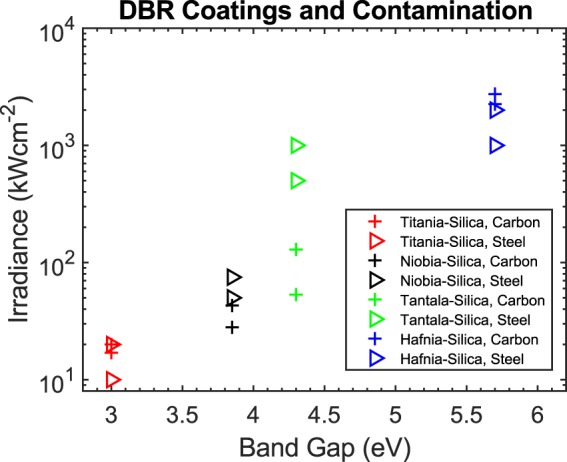
Laser-induced damage thresholds for microparticle carbon and stainless steel contaminated DBR coatings. Lowest damaging and highest survived irradiances are plotted for each DBR. Notice the clear trend of increasing damage thresholds with bandgap energy.

During testing, carbon contaminated distributed Bragg reflectors (DBR) coatings failed at irradiances as low as 17 kWcm^−2^ for titania-silica while hafnia-silica started to fail at 2.25 MWcm^−2^. When damage occurred to a DBR, the optic failed catastrophically with the laser boring several millimeters into the fused silica substrate before the laser could be shut off manually. Comparing the damage thresholds to the bandgap energy of each film, a clear trend of increasing damage thresholds with larger bandgap energy is readily apparent, see Fig. 3. Stainless steel contaminated DBRs also displayed a similar bandgap dependence.

**Figure 4:**
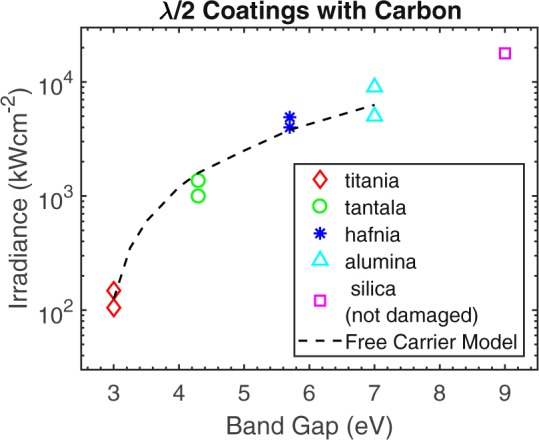
Laser-induced damage thresholds for microparticle carbon-contaminated λ/2 coatings. Lowest damaging and highest survived irradiances are plotted for each material. Silica λ/2 coatings (9 eV) were not damaged in testing. The dashed line represents thermally generated free carrier induced breakdown as predicted by our model.

The carbon contaminated half-wave (λ/2) coatings failed at irradiances as low as 105 kWcm^−2^ for titania films while silica coatings all survived the maximum tested irradiance of 17.8 MWcm^−2^. All materials closely followed the bandgap trend over all the test irradiances, see Fig. 4. When damaged, the top film layer cracked, delaminated, or was removed completely from the underlying substrate though in most cases damage was self limiting and no deeper substrate damage occurred.

With the bandgap dependence observed, free carrier generation and absorption were hypothesized as the most likely processes ultimately responsible for early failure; however, there are several possible mechanism for generating free carriers. Thermal free carrier generation processes will be discussed in the next section. One other potential free-carrier generation mechanisms was photoexcitation from the UV Planck emission of evaporating contaminant particles. However the testing of UV with high power IR illumination failed to damage any pristine coatings despite testing low bandgap materials with over 50 kWcm^−2^ of 224 nm and up to 13 MWcm^−2^ of 1070 nm light, see Table 1. These same optics had failed at between 17 and 20 kWcm^−2^ when contaminated with carbon. Alignment of the two beams was carefully checked and several tests were done moving the sample through the focus of the UV laser as to ensure the maximum UV irradiance occurred during the test. Initially some random damage events happened during setup, though these were traced to airborne contamination left over from an unrelated experiment. After all the lab surfaces were wiped down with isopropyl alcohol and the room air filtered, no more damage events occurred in the clean conditions. The UV irradiance produced by the HeAg laser is orders of magnitude greater than what could be generated from Plank emission of a superheated surface particle. The fact that this level of UV irradiance did not cause any failures despite exposing samples to 13 MWcm^−2^ of near-infrared power, three orders of magnitude greater than the damage threshold with carbon contamination, effectively rules out UV photo-excitation as the damage mechanism for the contaminated optics.

**Table 1:** Number of tests of IR + UV illumination for each HR coating type.

DBR Type	100 kWcm^−2^	250 kWcm^−2^	500 kWcm^−2^	1 MWcm^−2^	2 MWcm^−2^	3 MWcm^−2^	3.4 MWcm^−2^	5 MWcm^−2^	13 MWcm^−2^
Titania-Silica							3 tests		
Niobia-Silica	1 tests	1 test	1 test	2 tests	2 tests	1 test	4 tests	1 test	16 tests
Tantala-Silica				1 test				1 test	1 test

No damage from the combination of IR and UV exposure occurred even at the highest test irradiances. With carbon contamination titania-silica HR materials began failing at 17 kWcm^−2^, niobia-silica at 28 kWcm^−2^, and tantala-silica at 53 kWcm^−2^.

**Figure 5:**
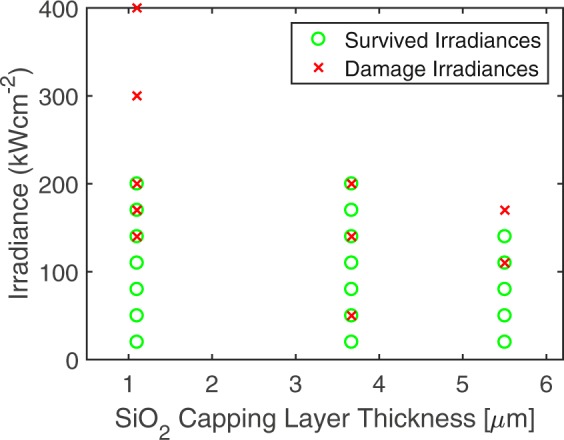
Laser damage tests of carbon contaminated tantala-silica DBRs with protective silica capping layers. There is no appreciable increase in the damage threshold for the thicker capping layers.

The samples with silica capping layers showed little to no improvement in damage thresholds over normal uncapped DBRs, see Fig. 5. This originally seemed at odds with any model involving direct thermal conduction from particle to substrate; however, the thermal model of laser damage developed in the next section shows that the capping layer thickness was too thin to effectively insulate the low bandgap materials from the superheated surface contamination. Results of experiments where the substrates were contaminated on the backside and illuminated from the backside showed no failures up to the highest powers attainable in our testing. Here the capping layer thickness was effectively that of the entire substrate.

### Thermally Generated Free Carrier Model

Since free-carrier generation via heated surface contamination was consistent with our initial data (once the effects of capping layers and UV illumination were understood), we constructed a physical model to determine if it could predict the quantitative breakdown values that were experimentally observed. In this model, laser radiation is absorbed by a contaminant particle on the surface of an optical coating. The absorbed laser energy causes the particle temperature to rise to sublimation, and the particle begins to lose mass to evaporation. During this period, heat from optical absorption and the particle-film interface thermally generates free carriers in the film. If a sufficient concentration of carriers is generated, the subsequent free-carrier absorption creates a runaway thermal breakdown. However if the particle fully evaporates before sufficient free carriers are generated, heat ceases to be transferred into the coating and the optic will survive.

The optical absorption of the sample and the thermal contact conductance (TCC) between the particle and film are the key parameters that determine the heating of the coating. The absorption of carbon contaminated samples measured by PCI varied by location, with values of tens of thousands being typical, see Fig. 1. In the model an average value of 20 000 ppm was used. TCC values were previous calculated at 46 kWm^−2^K^−1^ during laser conditioning experiments of 1 µm graphite flakes^14^. To gain an understanding of the meaning of this value, typical metal-metal mechanical contacts generally have a TCC on the order of a few kWm^−2^K^−1^ ^15^. This low value indicates both the roughness and the weak incidental bonding of surfaces temporarily in mechanical contact. At the other extreme, the TCC of graphene and highly ordered graphite can easily be tens of MWm^−2^K^−1^ ^16^. Due to the loose, disordered nature of the graphite flakes in our study, a value much lower than highly ordered graphite but somewhat larger than metal-metal contacts makes physical sense. The 7 µm carbon particles used in laser damage testing are relatively close in size and shape to the 1 µm graphite flakes for which the TCC estimate was calculated and for the model a TCC of 48 kWm^−2^K^−1^ was found to best fit the experimental data. This value is so close to the value measured by direct TCC measurements^14^ that it is considered to be a strong indicator of the validity of the model.

Heat losses from radiation, air convection and air conduction had to also be considered. Radiation losses emitted upwards from the particle and film surface were calculated by the Stefan-Boltzmann law and were included in the simulation, though their effect was minor. Coefficients for free air convection are typically less than 10 Wm^−2^K^−1^ ^17^, three orders of magnitude smaller than the estimated contact conductance between the particle and film and can safely be discounted. Air conduction with a thermal conductivity of 0.026 Wm^−2^K^−1^ and typical boundary layers of millimeters^18^ is also three orders of magnitude smaller than the contact conductance, and was also discounted.

**Figure 6:**
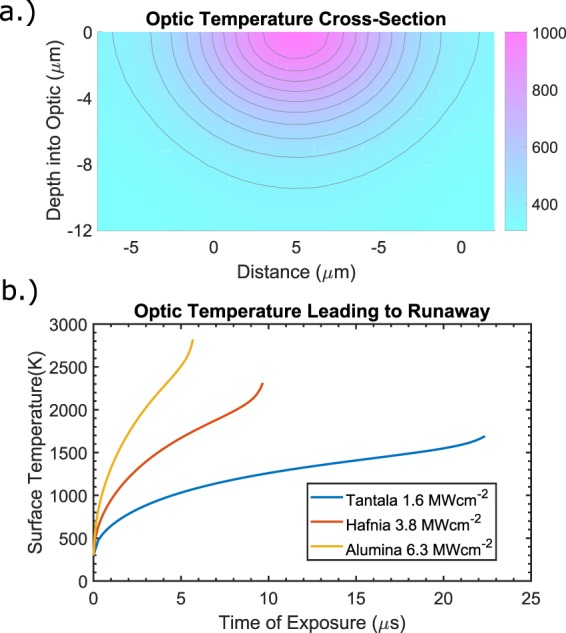
(**a**) Temperature profile of a tantala λ/2 coating at 1.2 MWcm^−2^ the moment the carbon contaminant fully evaporates. (**b**) Simulated surface temperatures of tantala, hafnia, and alumina coatings at their damage thresholds. Note the exponential increase in temperature near the end of each simulation as free carrier absorption begins to dominate. At this point the material will continue to absorb laser energy and thermally runaway even if the particle finishes evaporating.

To calculate the heat flux from the particle into the film via contact conductance, the temperature of the particle must be known in addition to that of the film. The evaporating particle is assumed to stabilize at its sublimation temperature of 4000 K^19^, as previous studies with evaporating contaminants have shown the temperature gradient across such a particle to be minimal^20^. Modeling heat flux between the particle and surface was done using MATLAB’s partial differential equation toolbox. The particle-film interface is defined a Neumann boundary condition, the heat flux crossing it determined by the optical absorption of the incident laser and the particle-film temperature difference with the estimated TCC. Heat entering through the boundary then diffuses downwards and outwards into the deeper layers of the film, see Fig. 6a. Free carrier absorption is included as a bandgap and temperature-dependent source term that adds heat within the film when sufficient carriers are generated, see equation 1

1 $$\frac{\partial T}{\partial t}=D{\nabla }^{2}T+\frac{{\alpha }_{FC}\,\ast \,{I}_{laser}}{\rho C}$$

D is the thermal diffusivity, α_FC_ is the free carrier absorption coefficient as a function of temperature and bandgap, I_laser_ is the irradiance of the incident laser, ρ is the density, and C is the heat capacity. To calculate the free carrier absorption, the Drude-Lorentz model of an electron plasma is used to estimate the imaginary part of the dielectric constant, which is then related to the absorption coefficient, see equation 2 ^21^.

2 $${\alpha }_{FC}=\frac{N{e}^{2}}{{m}^{\ast }{{\epsilon }}_{0}nc\tau }\,\frac{1}{{\omega }^{2}}$$

N is the density of free carriers, τ the phonon lifetime, and ω is the angular frequency of the incident light. The effective mass, m*, is taken to be the free space electron mass as the amorphous films lack the band curvature in k-space. The density of carriers N is simply calculated from density of states and the Maxwell Boltzmann distribution of energies, see equation 3 ^22^.

3 $${\rm{N}}=\sqrt{{N}_{c}{N}_{v}}{e}^{\frac{-{E}_{G}}{2{k}_{B}T}}$$

N_c_ and N_v_ are the effective density of states of the oxide band edges, and E_G_ is the material bandgap. Data for the effective densities of states in amorphous insulators is very sparse. Experiments with X-ray emission and quantum yield spectra find that amorphous SiO_2_ and α-quartz share very similar electronic structure^23,24^. Using numerical calculations based on the Hartree-Fock and density functional theory, a density of states of 3.5 × 10^22^ cm^−3^ was found for α-quartz^25^. Due to a lack of information about the other coating materials, the density of states for SiO_2_ were used throughout. For amorphous materials it is general practice to assume thermal transport is a series of random walks due to the disordered nature of the material. This results in an average phonon mean free path equal to the atomic spacing^26^. Dividing this by the speed of sound in the material gives the average phonon lifetime τ. Phonons also contribute to a reduction of bandgap energy. Disorder or variations of bond lengths within a material, give rise to extended tail states at the band edges, narrowing the bandgap^27^. At high temperatures the contribution to disorder from phonons exceeds that of the frozen-in disorder of the amorphous material^28^. This bandgap reduction was incorporated into the free carrier calculation by using experimental data from fused silica and scaling with respect to the relative bandgap energy.

The rate at which the contaminant is depleted can be found by comparing the power entering the particle from the absorbed laser irradiance to the power leaving the particle by thermal contact with the surface. The excess absorbed power not entering the surface goes into evaporation, see equation 4.

4 $${I}_{laser}\,\ast \,{A}_{particle}={H}_{vap}\frac{\partial m(t)}{\partial t}+{A}_{particle}\,\ast \,TCC\,\ast \,({T}_{particle}-{T}_{surface}(t))$$

A_particle_ is the cross sectional area of the contaminant, H_vap_ is the heat of vaporization, TCC is the thermal contact conductance, T_particle_ is the particle temperature which we have assumed to be its sublimation temperature, and T_surface_ is the temperature at the surface of the film. Though integrating the equation 4 yields the evaporation time of the particle, in practice this was not possible numerically in MATLAB’s partial differential equation toolbox as equation 4 depends on the surface temperature of the film given by equation 1. For large irradiances (>100 kWcm^−2^), the energy transferred through thermal contact is significantly smaller than the absorbed laser power, and the particle mass rate can be directly calculated from the irradiance, see equation 5.

5 $$\frac{\partial m}{\partial t}=\frac{{I}_{laser}\,\ast \,{A}_{particle}}{{H}_{vap}}$$

For simplicity the cross sectional area of the particle is assumed to remain constant as the particle evaporates though in practice images of the residue left behind from removed particles are often slightly larger^14^. The simplification used to obtain equation 5 is not valid for the lower irradiances at which many of the DBR coatings failed. To accurately predict this behavior, future models will need to capture this transfer of energy without the assumptions used in equation 5.

When the entire volume of the particle has been vaporized, the heat flux from the particle into the underlying material vanishes, and the surface will begin to cool and the model optic will survive. If, however, sufficient surface temperatures are reached before the particle has fully evaporated, the free carrier absorption will begin to dominate the heat transfer, and the near-surface material will thermally runaway, see Fig. 6b.

For a given irradiance and bandgap, equation 1 was solved numerically using MATLAB, and laser damage was determined to occur if the surface temperature climbed exponentially due to free carrier absorption dominating before the particle had fully evaporated, determined by equation 5. This was repeated to find the minimum irradiance required to damage each bandgap energy. With the optical absorption and TCC values discussed previously, a close match to the experimental data was found, see Fig. 4.

The thermal diffusion model explains the observed ineffectiveness of the silica capping layers in preventing laser damage. At the irradiances that caused damage to the capped tantala-silica DBRs, the carbon contamination takes roughly 1 ms to fully evaporate. This is sufficient time for heat to conduct the several microns through the cap layer and into the lower bandgap tantala, where it can generate free carriers and cause damage. For a capping layer to be effective, it must be substantially longer than the length heat will diffuse during the evaporation time of particle contaminant, which for the example of tantala-silica DBRs would have been about 10 µm, twice that of the thickest capping layer tested. Backside illuminated samples have millimeters of silica that heat must diffuse through in order to reach the lower bandgap materials. This effectively insulates them from the particle heating and prevents any thermal generation of free carriers.

## Discussion

The strong bandgap dependence of damage thresholds seen for all of the optical materials and contaminants is an interesting and unexpected result. Contamination has long been known to cause early failures optics under intense illumination^5,11,29–31^, however the seemingly random nature of failure has frustrated efforts understand the exact process or to even define a clear damage threshold for a material^32^. This perceived randomness has caused particle-induced breakdown to be treated statistically often without a physical basis and where probabilities of failure gradually increase with irradiance^32^. Differences in the composition of the contamination, the average size and shape of the contaminants, and wide variations in thin film properties due to different deposition techniques and conditions all further add to the observed spread of damage thresholds. Though some particle/defect-induced breakdown models have been developed, they typically analyze thermal shock and film stress caused by uneven heating^6,33,34^. In our study, while heat transfer into and within the material is certainly important, none of the standard thermal parameters of thermal conductivity, melting point, and intrinsic film absorption fit the observed damage thresholds, see Table 2. Only differences in material bandgap are consistent with experimental observations. These observations show that low bandgap materials are a significant liability in high power optics if contamination is possible.

**Table 2:** Material properties of the films tested listed in the order which they damage.

Film	Absorption (ppm) and Film Type	Thermal Conductivity (Wm^−1^K^−1^)	Melting Point (K)	Bandgap (eV)
Titania	3.5 ppm λ/2	3	2048	3
Niobia	2 ppm DBR	1.5	1793	3.85
Tantala	12ppm λ/2, 0.7 ppm DBR	3	2191	4.3
Hafnia	35 ppm λ/2, 20 ppm DBR	1.2	3031	5.7
Alumina	30ppm λ/2	1.1	2345	7
Silica	1.5 ppm λ/2	1.1	1996	9

The absorption before contamination, thermal conductivity, melting point, and bandgap energy are listed. Note of these parameters, only the bandgap follows the observed damage sequence of the materials tested, monotonically increasing as the materials better resist damage^35–42^.

In contrast to prior statistical treatments, our model is deterministic in describing the laser damage process. For a given particle, optical material survival is determined by the bandgap of the material, the size of the contaminant that dictates the evaporation time, the optical absorption of the sample, and the TCC between particle and material. Statistics still play a role since higher contamination densities are more likely have particles or groups of clumped particles with sufficient mass and TCC to generate breakdown, but for each particle damage is simply a function of the above factors. Engineers designing optics for high-power systems should choose high bandgap materials and optical coatings for the top several microns of the optical surface in order to avoid contamination-induced failure.

## Conclusion

Contamination-induced breakdown is found to be a physically deterministic process that depends strongly on the bandgap of the materials underlying the contamination. This trend was observed for both λ/2 and DBR coatings and for both carbon and stainless steel contamination. Free carriers generated by interface thermal contact between the evaporating surface contaminants and the optical films/substrates are found to be the primary mechanism initiating failure. A physical model of heat transfer and free carrier absorption was developed that fits the experimental data with expected values for the absorption and thermal contact conductance. Engineers designing optics for high-power systems should choose high bandgap materials and optical coatings for the top several microns of the optical surface in order to avoid contamination-induced failure.
